# 2460. Understanding acquisition of *Candida glabrata* in the healthcare setting

**DOI:** 10.1093/ofid/ofad500.2078

**Published:** 2023-11-27

**Authors:** Jerald Cherian, Nathan Kwon, Lisa Maragakis, Patricia J Simner, Sara E Cosgrove, Clare Rock, Sean Zhang, Eili Klein

**Affiliations:** Johns Hopkins University School of Medicine, Baltimore, Maryland; Johns Hopkins University, Germantown, Maryland; Johns Hopkins Medicine, Baltimore, MD; Johns Hopkins School of Medicine, Baltimore, MD; Johns Hopkins School of Medicine, Baltimore, MD; Johns Hopkins School of Medicine, Baltimore, MD; Johns Hopkins Hospital, Baltimore, Maryland; Johns Hopkins School of Medicine, Baltimore, MD

## Abstract

**Background:**

Given increasing resistance and incidence of *Candida* infections in acute care hospitals, it is critical to understand the epidemiology of healthcare-associated acquisition of these fungal pathogens. Electronic health record (EHR) data is an underutilized resource in identifying potential transmission in this setting.

**Methods:**

Retrospective analysis of potential source of hospital-onset infections (HOI) with *Candida glabrata* ( >48 hrs after admission) between 1/1/2018 and 2/29/2020. Patient location in the hospital and documented contacts between patients and healthcare personnel (HCP) were extracted from the hospital EHR system. Documented contact by a HCP with 2 different patients within 6 hours was considered a potential connection between patients. Prior room occupancy within 7 days was considered potential environmental source. Similarity of *C. glabrata* infection was determined by isolate antifungal susceptibility profiling where minimum inhibitory concentration (MIC) to fluconazole and micafungin had to be within one doubling dilution difference.

**Results:**

A total of 156 *C. glabrata* infections were identified, of which 104, were considered HOIs. Of the 104 HOIs, 16 (15%) were associated with HCP connections, though 3 (3%) were also associated with prior room occupants. Four of the isolates matched exactly, while 7 had the same patterns of resistance and the MICs of fluconazole were within a single doubling dilution while micafungin was the same. One of the latter isolates was resistant to fluconazole. The mean time between HCP-associated infections was 11 days and the mean number of connections was 25.

Time-line of Candida glabrata infections and HCP-associated connections

**Figure:**
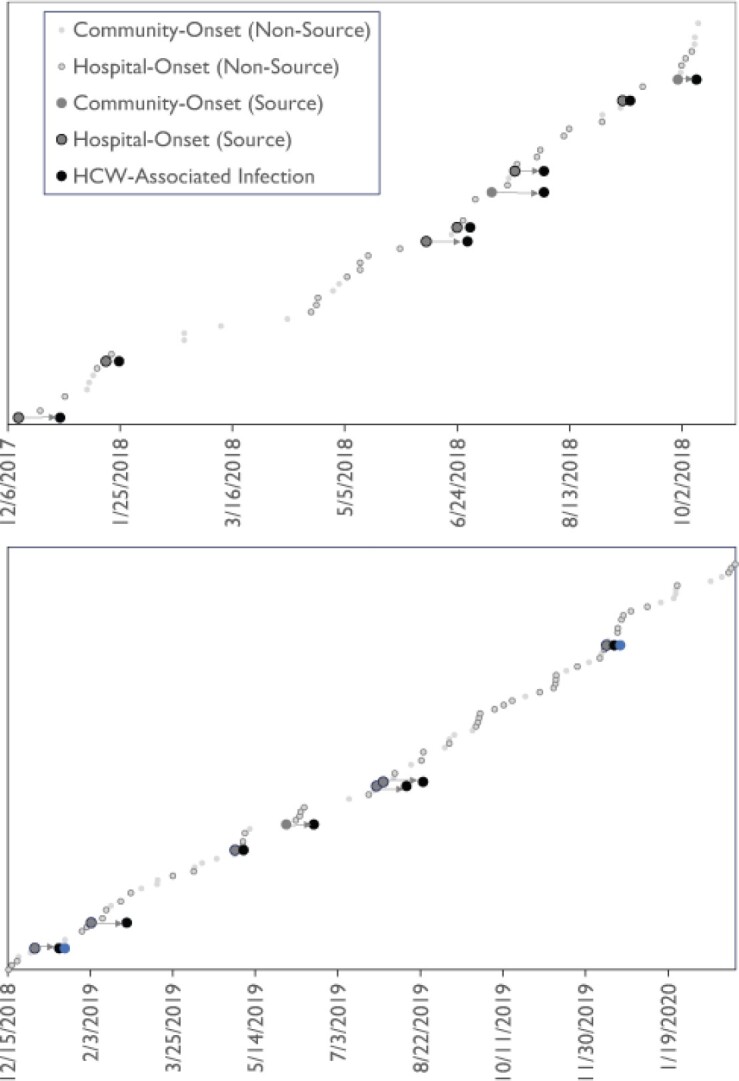

Top panel and lower panel are part of the same timeline but split into two panels for visualization. Dots denote collection date of C. glabrata isolates. Larger grey dots are isolates from potential source patients (i.e., had C. glabrata isolated prior to the target patient with whom they have a HCP-associated connection) while black larger dots are the target patients. In two cases there was suspected onward transmission from a secondary infection, denoted by the larger blue dots. Arrows denote the associated transmission. Highlighted dots denote community vs. hospital-onset primary infections.

**Conclusion:**

Transmission of *C. glabrata* leading to HOI is a rare event; however, HCP-mediated transmission was the main source of identified potential transmission events rather than direct environmental contamination. While antimicrobial stewardship, early identification, and prevention interventions should continue to be prioritized in Candida prevention programs, EHR data can be helpful in identifying potential transmission events. Further studies are needed to evaluate *C. glabrata* colonization pathways, as well as other *Candida* spp., such as *C. auris*, which may have different transmission characteristics.

**Disclosures:**

**Patricia J. Simner, PhD**, Affinity Biosensors: Grant/Research Support|BD Diagnostics: Advisor/Consultant|BD Diagnostics: Grant/Research Support|Entasis: Advisor/Consultant|GeneCapture: Stocks/Bonds|Merck: Advisor/Consultant|OpGen Inc: Board Member|OpGen Inc: Grant/Research Support|OpGen Inc: Honoraria|Qiagen Sciences Inc: Advisor/Consultant|Qiagen Sciences Inc: Grant/Research Support|Shionogi Inc: Advisor/Consultant|T2 Biosystems: Grant/Research Support **Sara E. Cosgrove, MD, MS**, Debiopharm: Advisor/Consultant|Duke Clinical Research Institute: Advisor/Consultant **Sean Zhang, MD, PhD**, Applied BioCode: Grant/Research Support|IMMY Diagnostics: Grant/Research Support|KARIUS: Advisor/Consultant|Pearl Diagnostics: Grant/Research Support|Scanogen: Grant/Research Support|T2 biosystems: Advisor/Consultant|Vela Diagnostics: Grant/Research Support

